# Antimicrobial Resistance among Pregnant Women with Urinary Tract Infections Attending Antenatal Clinic at Levy Mwanawasa University Teaching Hospital (LMUTH), Lusaka, Zambia

**DOI:** 10.1155/2021/8884297

**Published:** 2021-03-04

**Authors:** Kekelwa Inyambo Yeta, Charles Michelo, Choolwe Jacobs

**Affiliations:** ^1^School of Public Health, Department of Epidemiology and Biostatistics, University of Zambia, Lusaka, Zambia; ^2^Levy Mwanawasa University Teaching Hospital, P. O. Box 810034, Lusaka, Zambia; ^3^Strategic Centre for Health Systems Metrics & Evaluations (SCHEME), School of Public Health, University of Zambia, Lusaka, Zambia

## Abstract

**Introduction:**

Globally, there is a growing concern over antimicrobial resistance (AMR) which is currently estimated to account for more than 700,000 deaths per year worldwide. AMR undermines the management of infectious diseases in general especially in pregnancy where significant bacteriuria continues to be a serious cause of maternal and perinatal morbidity and mortality. We therefore aimed to determine the prevalence of AMR and the associated factors among pregnant women with urinary tract infections (UTIs) attending antenatal clinic at a selected hospital in Lusaka, Zambia.

**Methods:**

This was a hospital-based, cross-sectional study conducted between November 2018 and May 2019. Interviewer-administered questionnaire was used to assess the sociodemographic characteristics and behavioural characteristics. Laboratory tests were also conducted. Descriptive statistics of study participants were used to describe the characteristics of the respondents. Chi-square was used to assess the association between categorical variables. The logistic regression analysis was carried out to generate the adjusted odds ratio with 95% confidence interval.

**Results:**

Overall (*n* = 203), the prevalence of UTI was 60% (95% CI: 53.3%–66.7%). The most isolated bacteria were *E. coli* (59%) and *Klebsiella* (21%). The prevalence of AMR was found to be 53% (95% CI: 46.1%–59.8%). The drugs highly resistant to antimicrobials were nalidixic acid (88.3%), ampicillin (77.8%), and norfloxacin (58.5%), while the least resistant drug was chloramphenicol (20%). There were no important significant predictors to AMR among pregnant women observed in this study.

**Conclusion:**

We found high burden of AMR closely linked to observe high prevalence of UTI suggested in this small population. This suggests a need to develop integrated surveillance systems that aim for early and regular screening of pregnant women for UTI as well as concurrent determination of antibiotic susceptibility patterns. This is important to prevent complications that may endanger maternal and fetal health outcomes. Furthermore, further research is needed to explore reasons for this high prevalence of AMR including examining possible attribution to the misuse of drugs so as to inform, enforce, or adjust the prescription-only policies and enforce antimicrobial stewardship programs.

## 1. Introduction

It is estimated that, about 150 million people worldwide are diagnosed with urinary tract infections (UTIs) [1]. Urinary tract infections are among the most common bacterial infections encountered by both the general community and in hospitals, ranking the second commonest infection after respiratory infections [2]. In poor-resourced and tropical countries, UTIs are still the major source of morbidity and death [3] with an estimated annual global incidence of at least 250 million in developing countries [4]. Women are more susceptible to UTI when compared to men, and this is largely due to short urethra, absence of prostatic secretion, pregnancy, and easy contamination of the urinary tract with faecal flora [5]. About 50% of women will suffer from at least one urinary tract infection (UTI) during their adult life including during pregnancy. There are many different pathogenic microorganisms (bacteria, fungi, protozoa, and viruses) which cause UTIs among pregnant women. Among the bacterial pathogens, *E. coli* and other *Enterobacteriaceae* are the most and account approximately 75% of the isolates [4].

Antibiotics are among the most commonly used medications to treat UTIs globally and are of enormous importance to global health. Despite their importance, the sustained effectiveness of antibiotics is endangered by the development of resistance. The excessive and unnecessary use of antibiotics has been the main cause of antibiotic resistance [6]. Internationally, there is a growing concern over antimicrobial resistance (AMR) which is currently estimated to account for more than 700,000 deaths per year worldwide [7]. Antimicrobial resistance is a broad term that includes resistance to all antimicrobial agents. Antimicrobial resistance results in the therapeutic failure of standard treatment and longer duration of treatment, leading to an increased risk in the spread of infections [8]. One of the important risk factors for antibiotic resistance is the abuse of antibiotics by the public [9], while some studies have also reported that some pregnant women are ignorant of the management of common infections which results in AMR [10].

In Zambia, like many other countries, there is emerging evidence of AMR in several pathogens [11–13]. Despite the large number of antimicrobial agents available, UTIs have remained a significant problem among pregnant women in Zambia [14], particularly in Lusaka. Yet, evidence on the prevalence of AMR among pregnant women and the associated factors is limited. It is against this background that the study was carried out to determine the prevalence of AMR and associated factors among pregnant women with UTI and attending antenatal clinic at a selected university teaching hospital in Lusaka, Zambia.

## 2. Methods

### 2.1. Study Setting and Period

A hospital-based cross-sectional study based on quantitative approach was conducted from November 2018 to May 2019. The study was conducted at Levy Mwanawasa University Teaching Hospital, located in Lusaka, the capital city of Zambia. The selected teaching hospital serves as a referral centre with a total catchment population of approximately 800,000 in- and outpatients.

### 2.2. Study Participants and Sampling

This study focused on all pregnant women who were attending antenatal clinic at Levy Mwanawasa University Teaching Hospital. All pregnant women aged 18 years and above, attending antenatal clinic within the data collection period, were targeted for the study, and they were hence purposely selected. Participants consenting to participate in the study were included in the study. We excluded pregnant women who were on antimicrobial therapy for UTI two weeks prior to selection and who provided inadequate urine samples (less than 10 ml urine), whose urine specimens were collected more than 2 hours before receipt for laboratory diagnosis, with specimens submitted in leaking or dirty unsterile containers and specimens revealing growth of more than two types of bacteria on culture.

### 2.3. Study Design

This was a hospital-based, cross-sectional study conducted between November 2018 and May 2019. Interviewer-administered questionnaire was used to assess the sociodemographic characteristics and lifestyle data. Laboratory tests were also conducted. Descriptive statistics of study participants were used to describe the characteristics of the respondents. Chi-square was used to assess the association between categorical variables. The logistic regression analysis was carried out to generate the adjusted odds ratio with 95% confidence interval.

### 2.4. Sample Size

The sample size was determined by using the single size population proportion formula with an assumption of 14% prevalence of antimicrobial resistance, from a study by Behailu Deres et al. [14]. Therefore, for 0.14, *p*, 1.96 *Z*, 95% CI, *a* = 0.05, and a 10% nonresponse rate, a sample size of 203 participants was determined. A convenient sampling technique was used to enroll consecutive pregnant women attending antenatal care in the hospital during the study period. Patients who tested positive for UTIs were recruited until the expected number of participants of the study sample was attained.

The main outcome variable was antimicrobial resistance, in pregnant women, a binary variable. Independent variables included sociodemographic characteristics (age, marital status, residence, education level, and monthly income) and clinical characteristics (HIV status, history of urinary tract infection, and trimester).

### 2.5. Laboratory Procedures

Urine specimens were inoculated onto CLED (cysteine-lactose-electrolyte-deficient agar) and MacConkey's and blood agar plates (OXOID, Ltd, Basingstoke, UK) by using the streak method following the standard microbiological procedures. The plates were incubated at 37°C for 24 hours and then examined for significant growth. Diagnosis of UTI was based on the presence of ≥10^5^ colony-forming units per millilitre of midstream urine of one or two types of bacterial species. Specimens with more than two types of bacteria species were regarded as contamination, and sample collection was repeated. The identification of bacteria isolate was done using biochemical tests.

Antimicrobial susceptibility testing was performed for the bacterial isolates identified from urine cultures with significant growth by using the Kirby–Bauer disk diffusion method on Mueller–Hinton agar (Oxoid Ltd, Basingstoke, UK) according to the criteria set by the Clinical and Laboratory Standards Institute [15, 16] to determine the susceptibility patterns of the commonly used antibiotics.

The procedure for antimicrobial susceptibility testing was as follows. In brief, 4–6 morphologically identical colonies of bacteria from pure cultures were collected with an inoculating loop, transferred into a tube containing 5 mL of nutrient broth, then mixed gently until a homogenous suspension was formed, and incubated at 37°C for 3–5 hours until the turbidity of the suspension became adjusted to the density of 0.5 McFarland standards, which yields a uniform suspension containing 105–106 cells/mL.

Using a sterile nontoxic dry cotton swab, the sample of the standardized inoculums (turbidity was adjusted to obtain confluent growth) were taken and streaked on the entire surface of the dried Mueller–Hinton agar plate three times, turning the plate at 60° angle between each streaking to ensure even distribution. The inoculum was allowed to dry for 5–15 minutes with the lid in place. Using standard antibiotic disks (Oxoid) containing nalidixic acid (30 *μ*g), nitrofurantoin (300 *μ*g), norfloxacin (10 *μ*g), chloramphenicol (30 *μ*g), co-trimoxazole (25 *μ*g), cefotaxime (30 *μ*g), penicillin (10 *μ*g)), gentamicin (10 *μ*g)), erythromycin (15 *μ*g)), ciprofloxacin (5 *μ*g), ampicillin (10 *μ*g)), and vancomycin (30 *μ*g) were dispensed onto well-labelled inoculated MHA plates using the disc dispenser. Sterile antibiotic disks used were based on their availability at the laboratory at the time of the study. The plates were allowed to stand for few minutes and were incubated at 37°C for 24 hours within 15 minutes of applying. Antibiotic sensitivity was checked by measuring the zone of inhibition (zone of clearance) from the back of the plate to the nearest mm using a ruler or caliper. Sterile zone of inhibitions was recorded and used to establish if the bacterial isolates were resistant, intermediate, and susceptible using reference books and WHONET. Bacteria were reported as sensitive (S), intermediate (I), or resistant (R) to each of the antibiotics used in the test.

### 2.6. Data Processing, Quality Control, and Analysis

Data were collected by face-to-face interviews using a standard questionnaire to collect sociodemographic and lifestyle data from the participants. The questionnaire was designed in English and translated to Nyanja, a commonly used local language in Lusaka. The tool was pretested for validation. Upon completion of the interview, the participants were sent to the hospital laboratory with a request form. Instructions were given to them by a trained medical laboratory personnel on how to collect the urine specimen. Participants were advised to place 10–20 mL clean-catch midstream urine specimen into a sterile screw-capped, wide-mouthed, sterile disposable plastic container after signing the consent form [15]. Each sample bottle was labelled with date and time of collection and then immediately sent to the microbiology department for microscopy, culture, and antimicrobial susceptibility. A unique sample number was linked to the participant`s questionnaire which was in turn linked to confidential patient information.

Data collected were verified for completeness and were double-entered into the excel spreadsheet to ensure accuracy and reliability. Culture and biochemical tests were performed by a laboratory scientist using the standard operating procedures to ensure quality results. The American Type Culture Collection (ATCC) reference strains such as *Escherichia coli* (ATCC-25922), *Staphylococcus aureus* (ATCC-25923), and *Pseudomonas aeruginosa* (ATCC-27853) were used as quality control parameters of laboratory tests.

The culture results from the samples collected were used to calculate prevalence of UTI, to characterize the type of microbial growth (isolates), and to test for antimicrobial susceptibility test. The data generated from the questionnaires were entered and checked for completeness, consistency, and accuracy and then entered into an Excel spreadsheet. After manual verification and cleaning, the data processing and statistical analysis was performed using STATA software version 14.0 (Stata™ Corporation, Texas, USA).

Basic descriptive statistics (proportions and means) of study participants were used to describe the characteristics of the variables of respondents. Categorical variables were summarized in the form of numbers and percentages and presented in table format. Continuous variables such as age were assessed for normality assumptions using Q-Q plots. Statistics such as means and their respective standard deviations were reported. Chi-square test was used to assess statistical differences between categorical variables with significance level set at *p* < 0.05 and 95% confidence interval. Bivariate analysis was applied, and all the variables with a *p* value less than 0.05 were then entered into the logistic regression model to generate the adjusted odds ratio with 95% confidence interval. A *p* value less than 0.05 (*p* < 0.05) was considered statistically significant.

### 2.7. Ethical Consideration

This study obtained ethical approval from the University of Zambia Biomedical Research Ethics Committee (UNZABREC) (reference number: 002-09-18). Authority to conduct the study was also obtained from the National Health Research Authority in Zambia. We received written informed consent from the study participants. Confidentiality was maintained by omitting personal identifiers. Privacy was also maintained.

## 3. Results

### 3.1. Participant Description

A total of 203 pregnant women were included in this study. As shown in Table 1, most of the pregnant women were married with a proportion of 80. 9% (165/206). A majority of the study participants were in the age range of 25 to 29 years and 30 to 34 (59% and 28.8%) respectively. Most of these women came from low-cost areas (areas with high population density and low cost of living) having a proportion of 68.2% (131/206), and 52% (102/202) of the women had secondary education as the highest level of education achieved. Approximately 61% (125/202) of the women had a history of previous urinary tract infection, and half of them (50%) were in their second trimester.

### 3.2. Prevalence of Urinary Tract Infection

The prevalence of urinary tract infection among pregnant women who were attending antenatal clinic at Levy Mwanawasa University Teaching hospital was found to be 60% (95% CI: 53.3%–66.7%).

**Figure 1:**
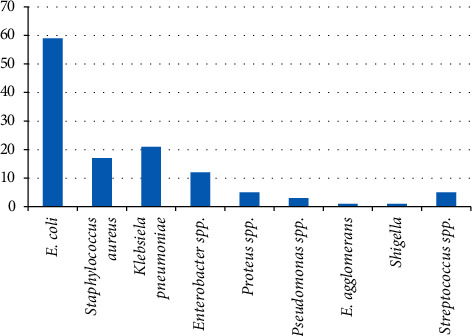
Prevalence of microorganism isolates.

The following bacterial uropathogens were identified: *E. coli*, 59 isolates (28%); *S. aureus*, 17 isolates (8.29%); *Klebsiella pneumoniae,* 21 isolates (10.24%); *Enterobacter spp.,* 12 isolates (5.85%); *Proteus spp.*, 5 isolates (2.44%); *Pseudomonas spp.,* 3 isolates (1.46%), and *Streptococcus spp.*, 5 isolates (2.44%), and the least isolates were *E. agglomerans,* 1 isolate (0.49%), and *Shigella*, 1 (0.49%) (Figure 1).

### 3.3. Prevalence of ANC-Based AMR

Out of 123 cases with significant bacteria growth, 65 (53%, 95% CI: 46.1%–59.8%) cultures plates were resistant to one or more drugs used in this study and 58 (47%, 95% CI: 46.1%–53.9) were susceptible to all the drugs.

**Figure 2:**
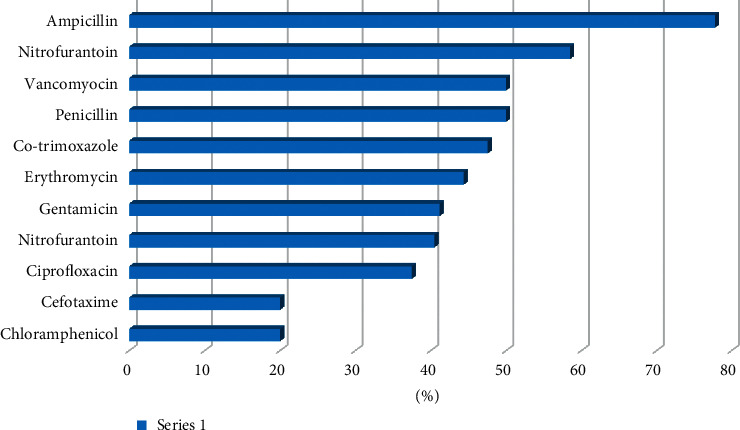
Resistance to antibiotics among pregnant women at LMUTH.

The prevalence of resistance in descending order was as follows: nalidixic acid, 88.3%; ampicillin, 77.8%; norfloxacin, 58.5%; vancomycin, 50%; penicillin, 50%; co-trimoxazole, 47.6%; erythromycin, 44.4%; gentamicin, 41.2%; nitrofurantoin, 40.5%; ciprofloxacin, 37.5; cefotaxime, 20%; and chloramphenicol, 20% (Figure 2).

### 3.4. Resistance Patterns according to Isolates

#### 3.4.1. Factors Associated with Antimicrobial Resistance

To determine the association between the dependent variable (antimicrobial resistance) and explanatory variables (age, current use of antibiotics, HIV status, duration of antibiotic use, symptoms, history of UTI infection, trimester, residence, and marital status), the univariate and multivariate binary logistic regression analyses were carried out. In the univariate analysis, history of antibiotic use (OR = 1.19, 95% CI: 0.38, 3.75; *p* < 0.770) had an increased likelihood of antimicrobial resistance compared to those who did not use antibiotics in the past. Similarly, the secondary and tertiary maternal education (OR = 1.95, 95% CI: 0. 64, 5.94; *p* < 0.24 and OR = 1.23, 95% CI: 0.39, 3.86; *p* < 0.73, respectively) had an increased likelihood of antimicrobial resistance than the maternal primary education, while married women had a decreased likelihood of antimicrobial resistance compared to those who were single (OR = 0.58, 95% CI: 0.20, 1.71; *p* < 0.32). However, all these findings were not significantly associated with AMR.

The results from the multivariate analysis showed that that current use of antibiotics reduced the likelihood of antimicrobial resistance compared to those not using antibiotics currently (AOR = 0.50, 95% CI: 0.13, 1.94; *p* < 0.31). Increased maternal age reduced the likelihood of antimicrobial resistance compared to women aged 18 to 24. The underlying and proximate determinants of antimicrobial resistance are shown in Table 1. From our study findings, it was noticed that most of the independent variables did not play a significant role in influencing antimicrobial resistance in pregnant women attending at antenatal at LMUTH (Tables 2–4).

## 4. Discussion

We have found the prevalence of urinary tract infection to be 60% among pregnant women with urinary tract infections (UTIs) attending antenatal clinic at a selected hospital in Lusaka, Zambia. The main causative pathogens for the UTIs were *E. coli* and *Klebsiella*. These results are consistent with studies conducted in Libya [17, 18] which found *E. coli* and *Klebsiella* as the highest isolated bacteria. The prevalence of AMR was found to be 53% with the highest resistant drugs being ampicillin, nalidixic acid, and norfloxacin. The least resistant were found to be chloramphenicol and nitrofurantoin. Our results of AMR are similar to a study conducted in Libya, [15] which found the AMR prevalence of between 10.5% and 64.5% with high levels of resistance to ampicillin, and Australia [19], with low levels of resistance for nitrofurantoin. Our study further determined both demographic and clinical predictors of AMR, which, however, showed no significant statistical association with the outcome variable. All the sociodemographic variables had *p* values greater than 0.05, similar to a study conducted in Ethiopia, [20] where they did not find any sociodemographic predictor variables for AMR, except for clinical and bacterial variables.

Urinary tract infections (UTIs) are the most widely spread infections seen in hospital settings and the second commonest infection seen in the general population [21]. The main findings of this study were prevalence of urinary tract infections, at 60%. These results are similar to other studies conducted in Ebonyi state, Nigeria [22], and in India [23] which had a prevalence of 55% and 61%, respectively. Our observation of the high prevalence found in this study highlights the burden of the problem which could be related to poor personal hygiene for some women as a result of vaginal anatomical and functional changes and challenges for some women in maintaining personal hygiene during pregnancy [24, 25] including the socioeconomic status of some women [26, 27]. Importantly, the high prevalence of urinary tract infections observed in this study is a concern due to the many adverse implications it has on pregnant women such as poor maternal and perinatal outcomes, sepsis, caesarean delivery, and preterm birth [28, 29].

We have also found that the three most prominent bacteria isolated among the participants were *E. coli* (28.78%), *Staphylococcus aureus* (8.29%), and *Klebsiella pneumoniae* (10.24%) of which *E. coli* was the most predominant. The least isolated bacteria were *Shigella* (0.49%) and *E. agglomerans* (0.24%). These results are comparable with studies carried out in Ethiopia [30], Libya [17], and Nigeria [31], which had higher isolates of *E. coli* and *Klebsiella*, although contrary to results from studies carried out in Brazil [32] and India [23] which showed *Staphylococcus aureus* as the most isolated bacteria. The similarities and differences in the type and distribution of uropathogens show a discrepancy from country to country due to many factors such as environmental conditions, health practices, patient conditions, personal hygiene, number of patients examined, and laboratory procedures [15]. Finding that *E. coli* is still the main causative agent of UTI in pregnant women could be explained by the fact that it is a normal flora in the lower gastrointestinal tract, although its implication is a concern as it causes diarrhoea, a threat for dehydration in pregnant women [33, 34].

The prevalence of the resistance of the different drugs was determined and indicated quite high level of resistance for drugs such as ciprofloxacin and chloramphenicol that are commonly prescribed. The observed high level of resistance could probably be because they have been on the market for a long time, thus allowing time for microorganisms to develop resistance mechanisms towards the antibiotics [35–37]. In addition to this, this level of resistance could be attributed to easy access to antibiotics over the counter in developing countries like Zambia [35]. Additionally, the initial use of antibiotics before the laboratory results of antimicrobial susceptibility can be an attribution to the high resistance levels. Consequently, the need for the development and enforcement of antibiotic policies and proper antibiotic stewardship in developing countries cannot be overemphasized.

Our study points out that nitrofurantoin was more susceptible to uropathogens than the most commonly prescribed drugs, and this is interesting considering the fact that there have been debates to have it phased out. Literature study reviews that there is a need to resurface old drugs as they would actually be more effective, some of these drugs such as nitrofurantoin and fosfomycin, i.e., specifically for resistant uropathogens [19].

Furthermore, determinants of antimicrobial resistance were analysed, and it was found that there was no statistical significance associating the socialdemographic and clinical variables with resistance. These findings are similar to a study which was carried out in Ethiopia [36] where they, as well, did not find a statistical association between the sociodemographic and clinical characteristics with resistance except for phenotypic and genotypic characteristics.

This study has limitations worth noting. Firstly, the study was liable to information bias due to social desirability and recall bias. However, this was controlled through validating medical records and through laboratory analysis. Secondly, we could not test all the drugs due to limited unavailability of some of the susceptibility drug discs as the research was not sponsored, hence limiting us to the drug discs available to the hospital at every given point.

However, although these limitations could have occurred, we think they did not significantly influence our findings. The results of this study remain important and add to the body of knowledge on the prevalence of AMR among pregnant women seeking antenatal care and associated factors in a low-resourced country like Zambia.

## 5. Conclusion

The UTI prevalence of 60% found among pregnant women in this study is generally much higher than most studies undertaken, which is indicative of the level of menace urinary tract infections in Zambia. This is alarming concern considering the adverse effects of untreated UTI on the mother and fetus such as kidney infections, preterm birth, low birth weight, and miscarriages. The highest causative agent of urinary tract infections was found to be *E. coli* which is another concern considering that it has the highest resistant strains which is resistant to the 3^rd^ generation cephalosporins implying that treatment of severe infections relies on carbapenems which are more expensive and might be a challenge for low-income countries. Furthermore, finding that the prevalence of antimicrobial resistance was at 53% with nalidixic acid, norfloxacin, and ampicillin being the most resistant is another concern considering that these drugs are commonly and routinely given, hence questioning treatment effectiveness.

Antimicrobial resistance results in this study additionally reflects the need for development of policies that restrict the usage of antibiotics and need to strengthen actions to ensure that antibiotics are used appropriately, such as re-enforcing prescription-only policies, implementing surveillance of antimicrobial consumption, and implementing antimicrobial stewardship programs [35].

Furthermore, finding that sociodemographic characteristics were not associated with antimicrobial resistance in this study suggests need for conducting similar studies such as [20] that include examining genotypic and phenotypic characteristics.

## Figures and Tables

**Table 1:** Sociodemographic characteristics of study participants at Levy Mwanawasa University Teaching Hospital (*n* = 203).

Characteristics	Total (*n* = 203)	
	Patients (*n*)	Percentage (%)
Marital status		
Single	36	17.6
Married	163	80.9
Separated	1	0.4
Widowed	1	0.4
Divorced	2	1.0

Age in years		
Less than 20	9	4.3
20 to 24	43	20.0
25 to 29	59	28.8
30 to 34	59	28.8
35 to 39	24	12.7
40 and above	9	4.4

Residence		
High cost	15	7.8
Low cost	131	68.2
Medium cost	46	23.0

Education level		
Primary	28	14.3
Secondary	102	52.0
Tertiary	66	33.7

Monthly income (USD)		
No income	48	23.4
10.1 to 15	42	20.5
15.1 to 20	26	12.7
20.1 to 30	50	24.4
Above 30	39	19.0

HIV status		
Reactive	74	36.8
Nonreactive	127	63.2

History of urinary tract infection		
Yes	125	61.0
No	78	39.0

Pregnancy trimester		
First	19	10.8
Second	88	50.0
Third	69	39.2

**Table 2:** Susceptibility patterns of *Staphylococcus aureus*.

Antibiotics tested	*Staphylococcus aureus* (*n* = 50)		*Streptococcus* spp. (*n* = 10)	
	Total	Resistance (*n* %)	Total	Resistance *n* (%)
Nitrofurantoin	5	2 (40%)	2	1 (50%)
Nalidixic acid	5	3 (60%)	0	0 (0%)
Chloramphenicol	4	1 (25%)	1	0 (0%)
Norfloxacin	5	3 (60%)	1	1 (100%)
Co-trimoxazole	7	3 (43%)	2	1 (50%)
Ciprofloxacin	6	1 (17%)	3	1 (33%)
Cefotaxime	1	0 (0%)	—	—
Gentamicin	6	3 (50%)	—	—
Penicillin	2	0 (0%)	—	—
Ampicillin	2	1 (50%)	—	—
Erythromycin	7	4 (57%)	1	0 (0%)

**Table 3:** Susceptibility patterns of Gram-negative isolates.

Antibiotics tested	*E. coli* (*n* = 126)		*Klebsiella* (*n* = 54)	
	Total	Resistance (*n* %)	Total	Resistance *n* (%)
Nitrofurantoin	38	14 (37%)	16	8 (50%)
Nalidixic	2	1 (50%)	17	14 (82%)
Chloramphenicol	15	3 (20%)	1	0 (0%)
Norfloxacin	27	14 (52%)	9	6 (67%)
Co-trimoxazole	7	4 (57%)	3	2 (67%)
Ciprofloxacin	15	7 (47%)	4	3 (75%)
Cefotaxime	9	2 (22%)	—	Drug not given
Gentamicin	7	1 (14%)	2	2 (100%)
Penicillin	2	1 (50%)	—	Drug not given
Ampicillin	3	2 (67%)	2	2 (100%)
Erythromycin	1	0 (0%)	—	Drug not given
Antibiotics tested	*Enterobacter* spp. (*n* = 29)		*Proteus* spp. (*n* = 17)	
	Total	Resistance (*n* %)	Total	Resistance *n* (%)
Nitrofurantoin	10	3 (30%)	4	1 (25%)
Nalidixic	10	9 (90%)	4	4 (100%)
Chloramphenicol	2	0 (0%)	1	0 (0%)
Norfloxacin	3	2 (67%)	4	3 (75%)
Co-trimoxazole	1	0 (0%)	1	0 (0%)
Ciprofloxacin	3	0 (0%)	—	Drug not given
Cefotaxime	—	Drug not given	—	Drug not given
Gentamicin	—	Drug not given	1	1 (100%)
Penicillin	—	Drug not given	—	Drug not given
Ampicillin	—	Drug not given	1	1 (100%)
Erythromycin	—	Drug not given	1	0 (0%)
Antibiotics tested	*Pseudomonas* (*n* = 18)		*E. agglomerus* (*n* = 3)	
	Total	Resistance (*n* %)	Total	Resistance *n* (%)
Nitrofurantoin	3	2 (67%)	1	1 (100%)
Nalidixic	3	2 (67%)	1	1 (100%)
Chloramphenicol	—	Drug not given	—	Drug not given
Norfloxacin	4	2 (50)	1	1 (100%)
Co-trimoxazole	7	3 (43%)	—	Drug not given
Ciprofloxacin	1	0 (0%)	—	Drug not given
Cefotaxime	—	Drug not given	—	Drug not given
Gentamicin	—	Drug not given	—	Drug not given
Penicillin	—	Drug not given	—	Drug not given
Ampicillin	—	Drug not given	—	Drug not given
Erythromycin	—	Drug not given	—	Drug not given

**Table 4:** Multivariant determinants of antimicrobial resistance.

Predictors	Crude odds ratio (95% CI)	*p* value	Adjusted odds ratio (95% CI)	*p* value
Age group				
18–24	1			
25–29	0.96 (0.32–2.81)	0.920	0.79 (0.17–3.72)	0.765
30–34	1.05 (0.35–3.18)	0.931	0.82 (0.14–4.69)	0.827
>35	0.44 (0.14–1.40)	0.163	0.15 (0.02–1.04)	0.055

Marital status				
Single	1	0.324	1	
Married	0.58 (0.20–1.71)		0.44 (0.07–2.65)	0.368

Education				
Primary	1		1	
Secondary	1.95 (0.64–5.94)	0.239	3.20 (0.55–18.4)	0.195
Tertiary	1.23 (0.39–3.86)	0.726	1.94 (0.28–13.7)	0.505

Trimester				
1^st^	1		1	
2^nd^	0.38 (0.10–1.47)	0.160	0.61 (0.11–3.34)	0.572
3^rd^	0.48 (0.12–1.93)	0.299	0.68 (0.10–4.72)	0.695

Residence				
High density	1		1	
Medium density	0.70 (0.28–1.73)	0.438	1.52 (0.35–6.52)	0.574
Low density	0.72 (0.26–1.98)	0.523	0.52 (0.10–2.79)	0.449

History of UTI				
No	1			
Yes	1.19 (0.38–3.75)	0.770	2.86 (0.13–61.2)	0.502

HIV status				
Negative	1		1	
Positive	1.29 (0.59–2.85)	0.524	2.60 (0.68–9.88)	0.162

Income				
Low (0–1500)	1		1	
Medium (1500–3000)	1.03 (0.44–2.40)	0.950	0.62 (0.14–2.80)	0.538
High (≥3000)	0.77 (0.26–2.32)	0.647	0.56 (0.07–4.27)	0.578

Kidney infection				
No	1		1	
Yes	0.27 (0.04–1.69)	0.161	0.29 (0.03–3.35)	0.321
Duration of antibiotic use				
7 days	1		1	
14 days	0.88 (0.33–2.31)	0.794	0.78 (0.20–3.06)	0.725

Current use of antibiotics				
No	1		1	
Yes	0.89 (0.35–2.30)	0.815	0.50 (0.13–1.94)	0.313

Symptoms				
Dysuria/nocturia/urgency	1		1	
Dysuria	1.11 (0.47–2.64)	0.816	1.00 (0.28–3.64)	0.998
Haematuria	1.16 (0.35–3.87)	0.805	1.78 (0.25–12.7)	0.565

## Data Availability

The data used to support the findings of this study are available from the corresponding author upon request.

## Abbreviations

ATCC: American Type Culture Collection
AMR: Antimicrobial resistance
CLED: Cysteine-lactose-electrolyte-deficient
CDC: Centres for Disease Control and Prevention
DDD: Defined daily dose
*E. coli*: *Escherichia coli*
GP: General practice
LMUTH: Levy Mwanawasa University Teaching Hospital
WHO: World Health Organization
UTIs: Urinary tract infections.
